# Molecular Cloning and Expression Analysis of *hyp-1* Type PR-10 Family Genes in *Hypericum perforatum*

**DOI:** 10.3389/fpls.2016.00526

**Published:** 2016-04-21

**Authors:** Katja Karppinen, Emese Derzsó, Laura Jaakola, Anja Hohtola

**Affiliations:** ^1^Genetics and Physiology Unit, University of OuluOulu, Finland; ^2^Climate laboratory Holt, Department of Arctic and Marine Biology, UiT the Arctic University of NorwayTromsø, Norway; ^3^NIBIO, Norwegian Institute of Bioeconomy ResearchÅs, Norway

**Keywords:** St. John’s wort, pathogenesis-related, PR proteins, defense response, gene expression, abscisic acid, salicylic acid, wounding

## Abstract

*Hypericum perforatum* L. is an important medicinal plant for the treatment of depression. The plant contains bioactive hypericins that accumulate in dark glands present especially in reproductive parts of the plant. In this study, pathogenesis-related class 10 (PR-10) family genes were identified in *H. perforatum*, including three previously unidentified members with sequence homology to *hyp-1*, a phenolic coupling protein that has earlier been suggested to participate in biosynthesis and binding/transportation of hypericin. The *PR-10* genes showed constitutive but variable expression patterns in different *H. perforatum* tissues. They were all expressed at relatively high levels in leaves, variably in roots and low levels in stem and reproductive parts of the plant with no specific association with dark glands. The gene expression was up-regulated in leaves after salicylic acid, abscisic acid and wounding treatments but with variable levels. To study exact location of the gene expression, *in situ* hybridization of *hyp-1* transcripts was performed and the accumulation of the Hyp-1 protein was examined in various tissues. The presence of Hyp-1 protein in *H. perforatum* tissues mostly paralleled with the mRNA levels. *In situ* RNA hybridization localized the *hyp-1* transcripts predominantly in vascular tissues in root and stem, while in leaf the mRNA levels were high also in mesophyll cells in addition to vasculature. Our results indicate that the studied *PR-10* genes are likely to contribute to the defense responses in *H. perforatum*. Furthermore, despite the location of the *hyp-1* transcripts in vasculature, no support for the transportation of the Hyp-1 protein to dark glands was found in the current study. The present results together with earlier data question the role of the *hyp-1* as a key gene responsible for the hypericin biosynthesis in dark glands of *H. perforatum*.

## Introduction

Pathogenesis-related (PR) proteins constitute of a large group of proteins in higher plants often associated in plant defense responses. Based on sequence homology and biological activities, these proteins are classified into 17 different families (van Loon et al., 2006; Agarwal and Agarwal, 2014). The PR-10 subfamily is the largest family with members reported in numerous plant species and it includes major food and tree pollen allergens (Radauer and Breiteneder, 2007; Fernandes et al., 2013; Nakamura and Teshima, 2013). The members of the PR-10 protein family share common features such as low-molecular weight (15–20 kDa) with typically acidic p*I*, similar three-dimensional structure as well as conserved P-loop region, and usually cytosolic location (Liu and Ekramoddoullah, 2006; Fernandes et al., 2013; Agarwal and Agarwal, 2014).

The biological significance of the PR-10 proteins is not well understood but they are proposed to have a wide range of roles in plants. Association of the PR-10 proteins in plant defense has been suggested since many of the proteins are induced or their expression is up-regulated under various biotic or abiotic stress conditions, and some members exhibit antimicrobial or ribonuclease activity (Liu and Ekramoddoullah, 2006; Fernandes et al., 2013; Agarwal and Agarwal, 2014). There are also several reports of the up-regulation of the PR-10 gene expression by plant hormones and other signaling molecules transmitting plant defense responses (Pulla et al., 2010; Takeuchi et al., 2011; Jain et al., 2012; Agarwal and Agarwal, 2014). Structural studies have implied that the role of PR-10 proteins could be related to the binding and transportation of various hydrophobic ligands involved in plant development and defense-related signaling (Radauer et al., 2008; Fernandes et al., 2009, 2013). Few PR-10 members have also been proposed to perform an enzymatic condensation reaction between the ligands they bind (Bais et al., 2003; Lee and Facchini, 2010).

Many plant species have been reported to contain more than one PR-10 protein (Schenk et al., 2009; Bahramnejad et al., 2010; Lebel et al., 2010; Xie et al., 2010; Lee et al., 2012). The significance of the multiple closely related genes in a single plant species is not clear but they may contribute to the diversification of functions between the PR-10 genes (Lebel et al., 2010). For example in peach, two Pru p 1 protein isoforms have been reported to differ in their RNA hydrolysis and ligand binding activities (Zubini et al., 2009). In lupin, birch, grapevine, and ginseng, the members of the PR-10 gene family showed variable expression patterns in various tissues or in response to stress conditions indicating functional diversification between the family members (Pinto et al., 2005; Schenk et al., 2006; Lebel et al., 2010; Lee et al., 2012; He et al., 2013).

*Hypericum perforatum* L., commonly known as St. John’s wort, is a herbaceous perennial plant that has received considerable interest due to its medicinal properties. The plant is widely utilized for the treatment of mild to moderate depression, and the efficacy of the plant crude extracts has been confirmed by several clinical and pharmacological studies (reviewed in Russo et al., 2014). The medicinal properties of the plant are attributed to secondary metabolites called hypericins and hyperforins that are accumulating in dark and translucent glands, respectively, in the aerial parts of the plant, especially in reproductive parts (Karppinen and Hohtola, 2008). There are also evidences supporting the biosynthesis of hypericins in the dark glands (Zobayed et al., 2006; Kornfeld et al., 2007; Karppinen et al., 2008; Košuth et al., 2011). To date, one PR-10 gene from *H. perforatum*, called *hyp-1*, has been described, and its function has been suggested to be related with the biosynthesis and binding/transportation of hypericin (Bais et al., 2003; Michalska et al., 2010) as well as plant defense under stress conditions (Košuth et al., 2013). The objective of the present study was to investigate the presence of PR-10 family genes in *H. perforatum*. Here we report molecular cloning and expression analysis of three previously unidentified *H. perforatum* cDNAs with sequence homology to *hyp-1* and genes encoding class PR-10 proteins of other species. The expression of the three *PR-10* genes along with *hyp-1* were examined in various *H. perforatum* tissues as well as following wounding and treatments with stress-related signaling molecules to assess their potential contribution in plant defense. Furthermore, the *hyp-1* expression was analyzed at protein and cellular levels in order to obtain more detailed information of its location in the plant.

## Materials and Methods

### Plant Material

The *H. perforatum* L. plants of Finnish origin were grown in field conditions in the Botanical Gardens of the University of Oulu, Finland. Tissue samples (stem, root, leaf, and flower bud) were collected from the plants at the early stage of flowering. The collected leaves were dissected into leaf margins that contained dark glands and into leaf interior parts that were free of dark glands. Immediately after excision, all tissues were frozen in liquid nitrogen and stored at -80°C until they were used for RNA isolation, protein extraction and the determination of hypericins. Alternatively, tissues were fixed overnight at 4°C in 4% (w/v) paraformaldehyde and 0.25% (v/v) glutaraldehyde in 0.1 M sodium phosphate buffer (pH 7.0) for *in situ* RNA hybridization analysis. For stress treatments, the leaves of the plants were either wounded or sprayed with solutions of stress-related phytohormones (±)-abscisic acid (ABA; Sigma, St. Louis, MO, USA) or salicylic acid (SA; Sigma). Concentrations of the phytohormones, 100 μM of ABA and 10 mM of SA, were selected based on previously reported studies (Bahramnejad et al., 2010; Pulla et al., 2010). Wounding of the leaves was carried out by making parallel incisions with a razor blade lengthwise on leaves. The leaf samples were collected at 0, 3, 6, 10, 24, and 48 h after each treatment, immediately frozen in liquid nitrogen and stored at -80°C until they were used for RNA isolation.

### Isolation of RNA and cDNA Preparation

Total RNA was isolated from different tissues of *H. perforatum* according to Jaakola et al. (2001). The cDNA was synthesized from the total RNA using SuperScript III reverse transcriptase (Invitrogen, Carlsbad, CA, USA) with random primers according to the manufacturer’s instructions. The cDNA was purified from contaminating genomic DNA by using the method described by Jaakola et al. (2004).

### Isolation of *H. perforatum PR-10* Genes

To isolate *H. perforatum PR-10* genes, previously identified plant *PR-10* family genes were aligned and degenerate oligonucleotide primers were designed based on identified conserved regions. Degenerate primers 5′-ARATHATHGARGGNGAYG-3′ (forward primer) and 5′-RRTAYTCYTCNACYTGYT-3′ (reverse primer) were used for amplification of *PR-10* genes from *H. perforatum* cDNA. PCR reactions were performed with DyNazyme^TM^ II DNA polymerase (Finnzymes, Espoo, Finland) under conditions: initial denaturation at 94°C for 4 min, followed by 7 cycles at 94°C for 1 min, 70°C for 3 min, ramp rate of 0,1°C/s to 36°C and 72°C for 2 min, followed by 35 cycles at 94°C for 1 min, 40°C for 2 min, and 72°C for 2 min, and final extension at 72°C for 5 min. The amplified PCR products were gel-purified using a Montage^®^ DNA Gel Extraction Kit (Millipore, Bedford, MA, USA) and ligated into a pGEM-T Easy vector (Promega, Madison, WI, USA). Sequencing was performed by using an ABI 3730 DNA sequencer (Applied Biosystems, Foster City, CA, USA) with a BigDye Terminator Cycle Sequencing Kit (Applied Biosystems). The 3′ and 5′ cDNA ends were isolated using a SMART^TM^ RACE cDNA Amplification Kit (Clontech, Palo Alto, CA, USA). The nucleotide sequences of *HpPR10.1* (*hyp-1*), *HpPR10.2*, *HpPR10.3*, and *HpPR10.4* were deposited to GenBank under accession numbers KU565780, KU565781, KU565782, and KU565783, respectively.

### Sequence Analysis

For alignment and phylogenetic analysis of the *H. perforatum* PR-10 sequences, amino acid sequences of previously characterized PR-10 family proteins of other species were obtained from GenBank and aligned with *H. perforatum* PR-10 sequences by using Clustal Omega program. A phylogenetic tree was constructed by using the neighbor-joining method with the MEGA software, version 6.06. The reliability of the tree was evaluated by a bootstrap analysis with 1000 replicates. The predicted protein molecular weight was calculated using Compute pI/Mw tool (ExPASy Server). Signal peptide prediction was carried out using online tools SignalP 4.1 Server (Petersen et al., 2011) and Signal-BLAST, and the prediction of transmembrane domains was performed by using TMHMM Server v 2.0.

### Relative Quantification by Real-Time PCR

Real-time quantitative reverse transcription PCR (qRT-PCR) analyses were performed with a LightCycler^®^ 480 instrument and software (Roche, Basel, Switzerland). The transcript abundance of the isolated *H. perforatum PR-10* genes was detected using a LightCycler SYBR Green I Master qPCR Kit (Roche). The PCR conditions were an initial incubation at 95°C for 10 min followed by 45 cycles at 95°C for 10 s, 60°C for 20 s, and 72°C for 10 s. The gene-specific primer sequences used for the qRT-PCR analysis are shown in **Table 1**. For relative quantification of the PCR products, glyceraldehyde-3-phosphate dehydrogenase (*GAPDH*; GenBank Accession No. GU014528) was employed as a control gene. The results were verified by using *18S rRNA* (GenBank Accession No. AF206934) as a control gene. The results were calculated with LightCycler^®^ 480 software (Roche), using the calibrator-normalized PCR efficiency-corrected method (Technical note No. LC 13/2001, Roche).

**Table 1:** Gene-specific primers used for quantitative reverse transcription PCR (qRT-PCR) analyses.

Gene	Primer sequence 5′–3′
*HpPR10.1* (*hyp-1*)	CAGGCTGTTTAAGGCATTGGTC (forward)
	GGGATGTCCATCAACGAAAGTG (reverse)
*HpPR10.2*	AGAAATCAAGGTCGGACAAGAG (forward)
	CGAGGAAACAAGACCATAGAAC (reverse)
*HpPR10.3*	GAGGAAATCAAGCTAGGGCAAG (forward)
	TGACGACGACTATTGCACACAC (reverse)
*HpPR10.4*	GGCACAGGAAGCAAGGGTAAG (forward)
	GGGTAAACAAGGCCACCTCAG (reverse)
*HpGAPDH*	ATGGACCATCAAGCAAGGACTG (forward)
	GAAGGCCATTCCAGTCAACTTC (reverse)

The specificities of the amplified qRT-PCR products were verified by a melting curve analysis. The obtained PCR products were further subjected to agarose electrophoresis, followed by gel extraction using a Montage^®^ DNA Gel Extraction Kit (Millipore) and sequenced as described above to confirm the amplification of the desired product.

### *In Situ* RNA Hybridization Analysis

Fixed *H. perforatum* tissues were embedded in paraffin, sectioned, de-paraffined and rehydrated as described earlier (Karppinen et al., 2008). Digoxigenin (DIG)-labeled *hyp-1* sense and antisense RNA probes were obtained by *in vitro* transcription from a linearized plasmid containing a fragment of *hyp-1* cDNA. For the plasmid construction, a 312-bp fragment from the coding region of *hyp-1* was amplified from *H. perforatum* cDNA by PCR with DyNazyme^TM^ II DNA polymerase (Finnzymes) using primers 5′-AGGCATTGGTCCTTGAACG-3′ (forward) and 5′-CAGGCTTGGGATGATAGGAG-3′ (reverse) under standard PCR conditions. The PCR product was gel-purified, ligated into a pGEM-T Easy vector (Promega), and sequenced as described above to confirm the amplification of the desired product. *In vitro* transcription of the probes was performed from the linearized plasmid with either T7 or SP6 RNA polymerase using a DIG RNA Labeling Kit (Roche) according to the manufacturer’s instructions.

*In situ* RNA hybridization analysis was performed as described previously (Karppinen et al., 2008) with the exception that the hybridization with the RNA probes was carried out at 54°C. The *hyp-1* sense probe was used in negative control sections. The sections were examined and photographed under a light microscope (Nicon Optiphot-2; Nikon Corporation, Tokyo, Japan) or scanned with a confocal laser scanning microscope (LSM-5 Pascal; Zeiss, Jena, Germany).

### Immunoblotting Analysis

Proteins were isolated from *H. perforatum* tissues using a method described by Karppinen et al. (2010). The protein concentration of the extracts was determined according to Bradford (1976), using bovine serum albumin (Sigma) as a standard. Samples containing 30 μg of proteins were separated with sodium dodecyl sulfate-polyacrylamide gel electrophoresis (SDS-PAGE), using 12% resolving and 3% stacking gels. The separation was conducted using a Mini-Protean II electrophoresis system (Bio-Rad, Hercules, CA, USA) at 200 V. After electrophoresis, the proteins were either visualized with Coomassie Brilliant Blue R-250 (Merck, Darmstadt, Germany) or electroblotted for immunodetection onto polyvinylidene difluoride (PVDF) membrane (Bio-Rad) by using a Mini Trans-Blot Electrophoretic Transfer Cell (Bio-Rad) at 100 V for 2 h. The immunological detection of Hyp-1 protein was performed as described previously (Karppinen et al., 2010). Intensities of each protein band were quantified using Quantity One software (Bio-Rad). Samples from three independent plants were employed for analyses.

### Production of Recombinant Hyp-1 Protein

The coding region of the *hyp-1* gene was amplified from *H. perforatum* cDNA by PCR, using forward primer 5′-CTATTTTAACATTTGGATCC**ATG**GCGGCGTA-3′ (the translation start codon is in bold and the *Bam*HI site is underlined) and reverse primer 5′-GCAAAGGGTACC**TTA**AGCGAAAACTTCAGGA-3′ (the translation stop codon is in bold and the *Kpn*I site is underlined) under standard PCR conditions. The PCR product was gel-purified and ligated into *Bam*HI/*Kpn*I site of a pQE30 expression vector (Qiagen GmbH, Hilden, Germany). The obtained recombinant plasmid was transferred into *Escherichia coli* host strain M15 [pREP4] (Qiagen). The *E. coli* cells were grown in Luria-Bertani liquid medium in the presence of ampicillin (100 μg mL^-1^) and kanamycin (25 μg mL^-1^) at 37°C until the *D*_600_ of the culture reached 0.6. The cells were induced by 0.5 mM isopropyl thio-β-D-galactoside (IPTG) for 4 h at 37°C. The recombinant Hyp-1 protein containing an additional hexahistidine tag at the N-terminus was purified from the *E. coli* cells as described previously (Karppinen et al., 2008).

### Determination of Hypericins

HPLC-DAD was used for the determination of hypericin, pseudohypericin, protohypericin, and protopseudohypericin from different *H. perforatum* tissues as described previously (Karppinen and Hohtola, 2008). Samples from three individual plants were employed for analyses.

### Statistical Analysis

Quantitative results of analyses of gene expression, protein levels and content of hypericins are presented in terms of means ± SEs of at least three biological replicates. The effects of stress treatments on gene expression were analyzed with Student’s *t*-Test by using SPSS Statistics program, version 22 (IBM, New York, NY, USA).

## Results

### Cloning and Sequence Analysis of *PR-10* Genes

In a search for *H. perforatum PR-10* genes, four different nucleotide sequences were obtained with a homology-based PCR-method designated to target conserved regions of the *PR-10* genes. The first sequence (*HpPR10.1*) was identified as *hyp-1* gene that was first described and indicated for hypericin biosynthesis in *H. perforatum* by Bais et al. (2003). The other three genes, named according to usual nomenclature as *HpPR10.2*, *HpPR10.3*, and *HpPR10.4*, were isolated in full-length and they showed 79, 80, and 80% sequence identity, respectively, at nucleotide level to *hyp-1* gene (**Table 2**). All the isolated genes had a coding sequence (CDS) of 480 bp predicted to encode protein of 159 amino acids with a calculated molecular mass of 17.75–17.84 kDa and a theoretical p*I* ranging from 5.54 to 6.16 (**Table 2**). These protein features coincide well with those typically reported for PR-10 family proteins (Liu and Ekramoddoullah, 2006; van Loon et al., 2006; Fernandes et al., 2013) and earlier for *hyp-1* (Bais et al., 2003; Michalska et al., 2010). The proteins are likely to be cytoplasmic as no signal peptides or trans-membrane domains were detected in their sequences.

**Table 2:** Characteristics of the sequences of *H. perforatum PR-10* genes and their coding sequence (CDS) identity to each other.

Gene	GenBank no.	Characteristics				Sequence identity at nucleotide level (%)^1^			
		CDS (bp)	Amino acids	Protein mass (kDa)	p*I*	*HpPR10.1*	*HpPR10.2*	*HpPR10.3*	*HpPR10.4*
*HpPR10.1* (*hyp-1*)	KU565780	480	159	17.84	5.54	100	79	80	80
*HpPR10.2*	KU565781	480	159	17.75	5.80		100	91	91
*HpPR10.3*	KU565782	480	159	17.77	5.89			100	90
*HpPR10.4*	KU565783	480	159	17.81	6.16				100

*^1^The values were obtained from sequence alignments on Clustal Omega. CDS, coding sequence*.

**FIGURE 1:**
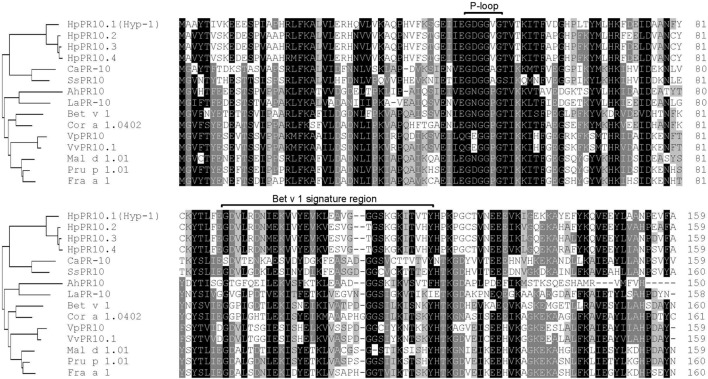
**Comparison of the deduced amino acid sequences of *Hypericum perforatum PR-10* genes with other PR-10 family proteins**. The conserved P-loop and Bet v 1 family motif signature region are showed in the alignment. A neighbor-joining tree based on the alignment is shown in the left. The GenBank accession numbers are as follows: *Arachis hypogaea* AhPR10 (AAU81922), *Betula pendula* Bet v 1 (CAB02159), *Capsicum annuum* CaPR-10 (AAF63519), *Corylus avellana* Cor a 1.0402 (AAG40329), *Fragaria* ×*ananassa* Fra a 1 (CAJ29538), *Lupinus albus* LaPR-10 (CAA03926), *Malus domestica* Mal d 1.01 (AAX18288), *Prunus dulcis* × *P. persica* Pru p 1.01 (ACE80940), *Solanum surattense* SsPR10 (AAU00066), *Vitis pseudoreticulata* VpPR10 (ABC86747), and *V. vinifera* VvPR10.1 (CAC16166). The HpPR10.1 (Hyp-1), HpPR10.2, HpPR10.3, and HpPR10.4 sequences have been deposited in GenBank under accession numbers KU565780, KU565781, KU565782, and KU565783, respectively.

Multiple sequence alignment analysis showed that the predicted amino acid sequences of the isolated *H. perforatum PR-10* genes (*HpPR10.1, HpPR10.2, HpPR10.3*, and *HpPR10.4*) had high homologies with other members of the PR-10 family proteins (**Figure 1**). All the four *H. perforatum* PR-10 sequences were found to contain a glycine-rich P-loop region (G-X-G-G-X-G) that is reported to be conserved among PR-10 proteins (Fernandes et al., 2013) and share similar Bet v 1 family signature motif region as described earlier for Hyp-1 by Bais et al. (2003). Phylogenetic analysis demonstrated that *H. perforatum* PR-10 sequences grouped as their own cluster similarly to other PR-10 proteins that also tended to cluster together with the homologs of the same taxonomic group (**Figure 1**). This type of clustering has been reported typical among PR-10 proteins and suggest gene duplication events during evolution (Radauer and Breiteneder, 2007; Lebel et al., 2010).

### Expression of *PR-10* Genes in *H. perforatum* Tissues

**FIGURE 2:**
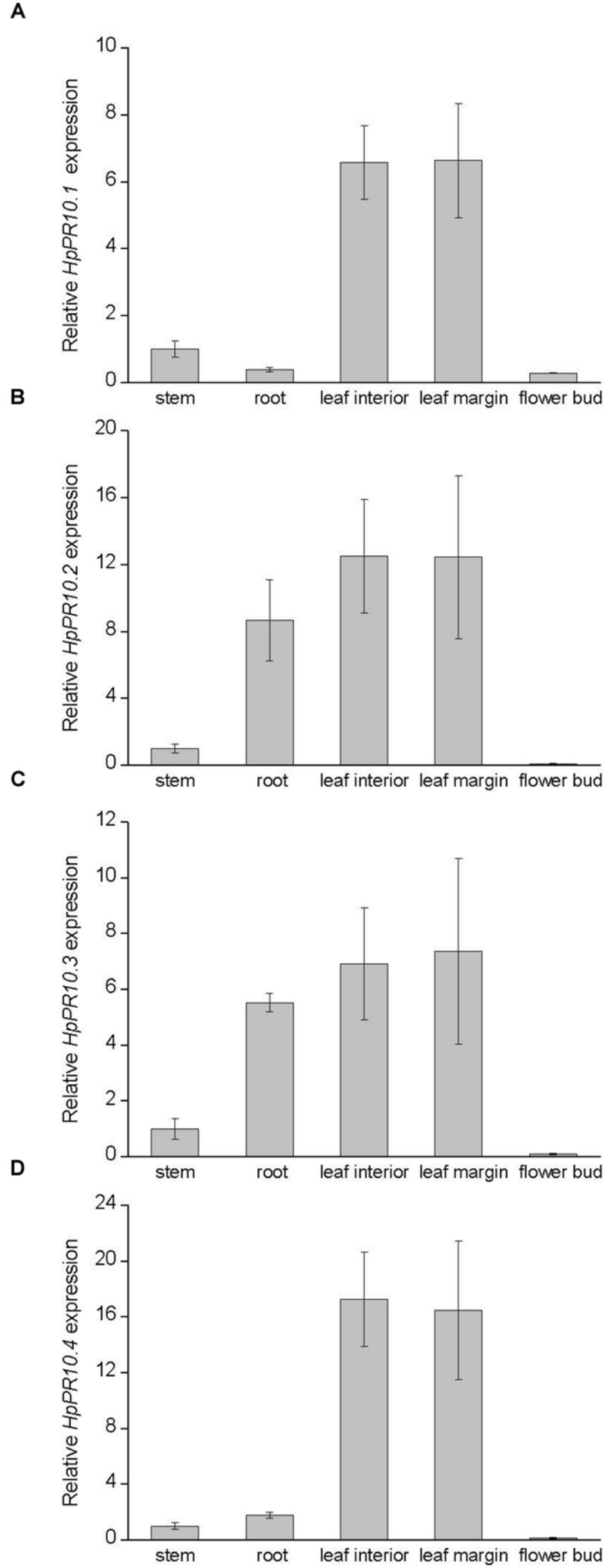
**Expression of *HpPR10.1* (*hyp-1*; **A)**, *HpPR10.2***(B)**, *HpPR10.3***(C)**, and *HpPR10.4***(D)** in *H. perforatum* tissues**. The relative expression of the genes was quantified by qRT-PCR and normalized to *GAPDH*. Values represent means ± SE of three biological replicates.

The transcript levels of the isolated *PR-10* genes were examined in different *H. perforatum* tissues with a qRT-PCR. All the genes were expressed at detectable levels in all tissues but with slightly variable expression patterns. Generally, the expression of all the genes was relatively high in leaf tissues, with no marked difference between leaf margin that contained dark glands and leaf interior part free of dark glands (**Figure 2**). Furthermore, the expression of all the genes was relatively low in stem tissue in comparison to leaf tissues and especially low in flower buds (**Figure 2**), the primary site for the accumulation of hypericins (**Supplementary Figure S1**). Instead, the expression levels of *HpPR10.1* (*hyp-1*; **Figure 2A**) and *HpPR10.4* (**Figure 2D**) were relatively low in root tissues while *HpPR10.2* (**Figure 2B**) and *HpPR10.3* (**Figure 2C**) had higher relative transcript levels in root. All the genes showed higher expression levels in younger parts of root closer to root tip compared to upper parts of root (data not shown).

### *In Situ* RNA Localization of *hyp-1*

**FIGURE 3:**
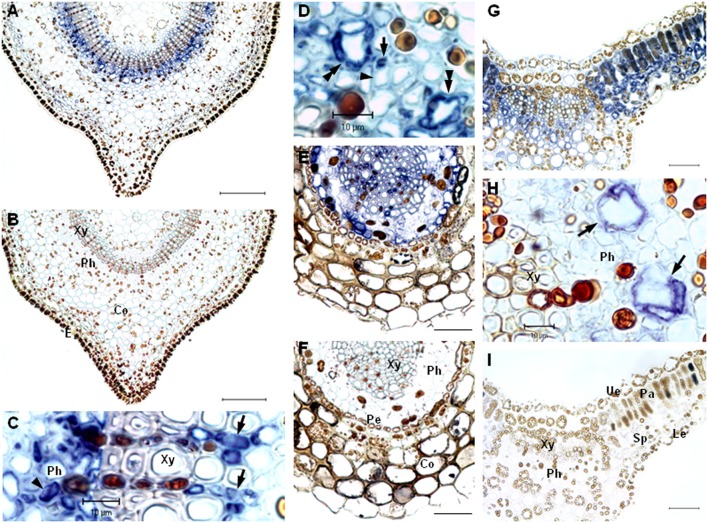
***In situ* localization of *hyp-1* transcripts in *H. perforatum* tissues**. Cross-sections of stem were hybridized with DIG-labeled RNA antisense probe **(A,C,D)** and sense probe **(B)**. The mRNA was detected abundantly in parenchyma cells of both xylem (arrows in **C**) and phloem (arrowhead in **C**) as well as in companion cells (arrow in **D**) and cells surrounding type A canals (double arrowheads in **D**). The expression was not present in sieve elements (arrowhead in **D**). Cross-sections of root hybridized with DIG-labeled RNA antisense probe **(E)** and sense probe **(F)**. Cross-sections of leaf hybridized with DIG-labeled RNA antisense probe **(G,H)** and sense probe **(I)**. The mRNA was present abundantly in mesophyll cells and cells surrounding type A canals in phloem (arrows in **H**). Xy, xylem; Ph, phloem; Co, cortex; E, epidermis; Pe, pericycle; Ue, upper epidermis; Pa, palisade parenchyma; Sp, spongy parenchyma; Le, lower epidermis. Bars = 50 μm, if not indicated otherwise.

The exact localization of the *hyp-1* gene expression in *H. perforatum* tissues was studied by *in situ* RNA hybridization. The study revealed that the *hyp-1* transcripts were mainly associated with leaf mesophyll as well as with the differentiated cells of vascular tissue in leaf, stem, and root. In stem, a blue signal for transcripts was mainly localized in both phloem and xylem cells in the area of vascular tissue but a weak signal was also present in the parenchyma cells under the stem epidermis (**Figure 3A**). The probe specificity was confirmed by the absence of any signal in the negative control sections of the stem hybridized with sense probe (**Figure 3B**). In the stem xylem, the signal was associated with xylem parenchyma cells in both the secondary and the primary xylem (**Figure 3C**). In the stem phloem, the signal was associated with parenchyma cells (**Figure 3C**) and small companion cells next to larger sieve elements (**Figure 3D**). The sieve elements showed no apparent signal. The mRNA was also apparent in cells surrounding specific secretory canals (**Figure 3D**), named type A canals earlier by Ciccarelli et al. (2001). In root, the transcripts were present in xylem parenchyma cells, in pericycle cells as well as in cells within the phloem (**Figure 3E**). No signal was detected in the corresponding areas of the negative control sections of roots (**Figure 3F**). In leaves, the mRNA was associated with both palisade and spongy parenchyma cells (**Figure 3G**). The signal was also detected in vascular tissues of leaves and was mostly associated with the cells surrounding the type A canals of the phloem (**Figure 3H**). No detectable signal was found in the cells of dark glands. Neither signal was detected in negative controls of leaf sections (**Figure 3I**).

### Immunoblotting Analysis of Hyp-1 Protein in *H. perforatum* Tissues

**FIGURE 4:**
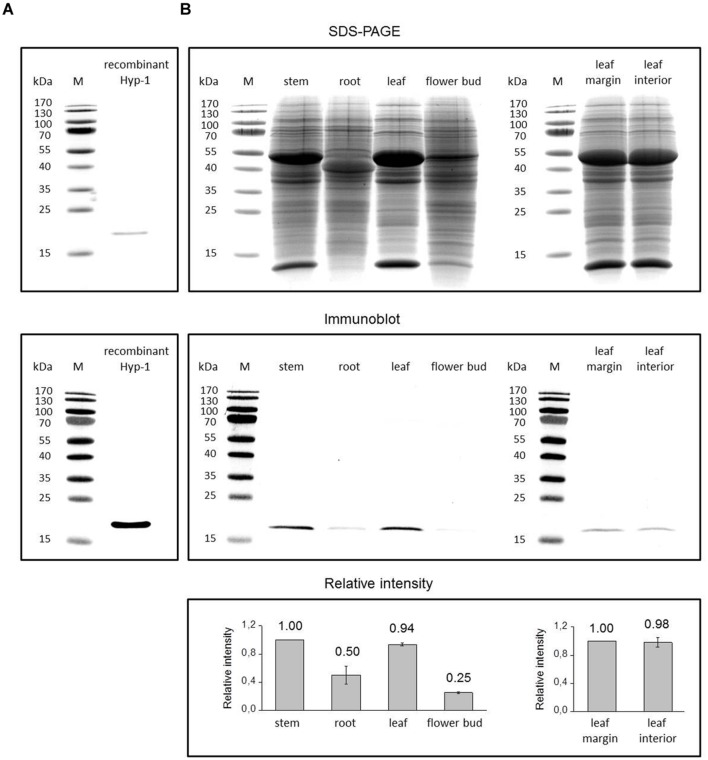
**Immunoblotting detection of Hyp-1 protein in *H. perforatum* tissues**. Coomassie Brilliant Blue stained SDS-PAGE gels and the corresponding immunoblots showing detection of Hyp-1 protein in samples of recombinant Hyp-1 protein **(A)** and *H. perforatum* tissues **(B)**. Lane M, protein molecular mass marker, with size (kDa) indicated on the left. Relative intensity values for protein levels in immunoblots represent means ± SE of three biological replicates.

We also examined the presence of Hyp-1 at protein level by immunoblotting analysis in the same *H. perforatum* tissues used for qRT-PCR analysis. In immunoblots, the antibody raised against Hyp-1 reacted with a purified recombinant Hyp-1 protein of about 18.5 kDa (**Figure 4A**) and a polypeptide of approximately 18 kDa in extracts of *H. perforatum* tissues (**Figure 4B**). The size coincides with the predicted molecular mass of 17.8 kDa for natural Hyp-1 protein that was calculated using bioinformatics tools and with the size that has previously been reported for Hyp-1 protein by other authors (Bais et al., 2003; Michalska et al., 2010). The small increase in the recombinant Hyp-1 protein size compared with the natural Hyp-1 protein is due to the presence of a His-tag at the N-terminus of the recombinant protein (12 additional amino acids). Immunoblotting analysis of *H. perforatum* tissues showed the highest level of Hyp-1 protein to be present in stem and leaf tissues while markedly lower levels were detected in root and especially flower buds (**Figure 4B**). Leaf margin containing dark glands and leaf interior part free of dark glands contained equal amounts of Hyp-1 protein (**Figure 4B**). The SDS-PAGE analysis demonstrated an equal loading of proteins to the gel with equal amounts of Rubisco subunits between the samples of leaf margin and leaf interior. The portion of the Rubisco subunits in the total protein loaded to the gel is high in stem and leaf samples but in flower buds and especially in root the Rubisco subunits form lower portion in the total proteins. This may cause some elevation in the level of Hyp-1 protein in immunoblot in these tissues relative to the green tissues.

### Expression of *PR-10* Genes in Response to Stress Treatments

**FIGURE 5:**
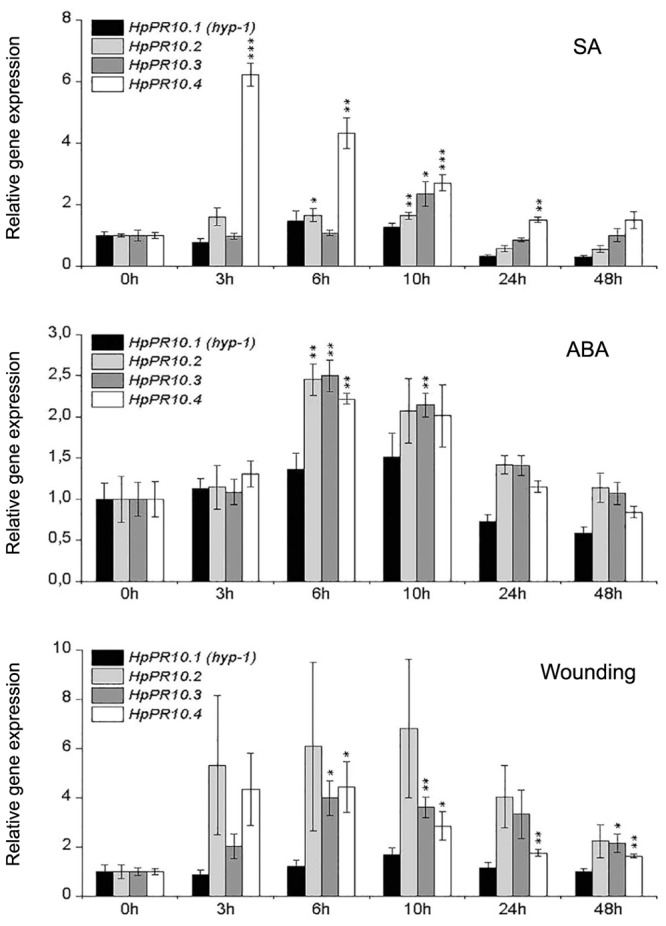
**Temporal expression patterns of *HpPR10.1* (*hyp-1*), *HpPR10.2*, *HpPR10.3*, and *HpPR10.4* in *H. perforatum* leaf tissues in response to treatments with SA (10 mM), ABA (100 μM) or wounding**. The relative expression of the genes was quantified by qRT-PCR and normalized to *GAPDH*. Values represent means ± SE of at least three biological replicates. Asterisks indicate statistically significant difference in comparison to untreated control (0 h) in Student’s *t*-Test at ^∗^*P* ≤ 0.05, ^∗∗^*P* ≤ 0.01, ^∗∗∗^*P* ≤ 0.001.

To examine whether the expression of the *HpPR10* genes are affected by different stress treatments, *H. perforatum* leaves were either wounded or treated with stress-related signaling molecules salicylic acid (SA; 10 mM) or abscisic acid (ABA; 100 μM). As shown in **Figure 5**, the treatment with SA significantly up-regulated the expression of *HpPR10.2*, *HpPR10.3*, and *HpPR10.4* in *H. perforatum* leaves. Especially the transcripts of *HpPR10.4* were highly induced by SA already 3 h after the treatment, and the expression gradually declined after that. The expression of *HpPR10.2* peaked at 6 h and *HpPR10.3* at 10 h after the SA treatment. Also the treatment with ABA significantly elevated *HpPR10.2*, *HpPR10.3*, and *HpPR10.4* expression after 6 h of the treatment with declining trend in the expression detected thereafter. Mechanical wounding of leaves significantly up-regulated the expression of *HpPR10.3* and *HpPR10.4* peaking 6 h after the treatment. Also *HpPR10.2* expression was elevated by the wounding but there seemed to be high variation between individual plants in the level of response to the treatment. None of the treatments significantly increased the expression of *HpPR10.1* (*hyp-1*) in *H. perforatum* leaves in the present study.

## Discussion

Many plant species have been found to contain several proteins belonging to the PR-10 family. Although the role of the PR-10 genes is not entirely known, functional diversification between the genes in plant development and protein-based defense has been suggested (Lebel et al., 2010). The presence of *PR-10* genes has also been reported in genus *Hypericum* (Bais et al., 2003; Jin et al., 2010). In the present study, the search for sequences encoding PR-10 proteins in *H. perforatum* revealed three previously unidentified members that were closely related to earlier described *hyp-1*, a phenolic coupling protein suggested to be involved in biosynthesis and binding/transportation of hypericin (Bais et al., 2003; Michalska et al., 2010). The isolated *H. perforatum PR-10* genes shared 79 to 80% identity at nucleotide level with *hyp-1*. The characteristics of the proteins, which were predicted to be small, acidic and cytosol-located, coincide well with those typically reported for PR-10 family proteins (Liu and Ekramoddoullah, 2006; Fernandes et al., 2013; Agarwal and Agarwal, 2014). Furthermore, their sequences contained features common to PR-10 family proteins, such as a glycine-rich P-loop conserved among PR-10 proteins (Fernandes et al., 2013) and shared similar Bet v 1 family signature motif region as described earlier for Hyp-1 (Bais et al., 2003).

The expression of some PR-10 proteins is known to be induced under certain stress conditions or expressed only in some tissues while some are constitutively expressed (Agarwal and Agarwal, 2014). Based on our results, all the studied *H. perforatum PR-10* genes were expressed in all analyzed tissues. Their expression was most highly associated with leaf tissues with lower transcript amounts found in stem and root tissues. Their expression differed from each other mostly in root tissue where expression of *HpPR10.2* and *HpPR10.3* was relatively high compared to relatively low expression of *HpPR10.1* (*hyp-1*) and *HpPR10.4* indicating possible specialization in their function between organs. We also found that the expression of all the *H. perforatum PR-10* genes was higher closer to the root tip. Earlier, Košuth et al. (2007) have reported that the *hyp-1* expression pattern of *ex vitro* plants differ from the pattern of young *in vitro* seedlings, which showed a high level of expression in roots. We have also demonstrated earlier that the developmental stage of leaf affects the presence of Hyp-1 protein (Karppinen et al., 2010) supporting the suggestion that the *H. perforatum PR-10* genes are likely to be developmentally regulated similarly to many other *PR-10* genes (Liu and Ekramoddoullah, 2006; Kim et al., 2008; Pulla et al., 2010).

The expression of the *HpPR10* genes was analyzed in this study for the first time in reproductive parts of *H. perforatum* which are rich with dark glands which form the primary accumulation sites of hypericins. All the genes were expressed relatively low levels in flower buds with no relation to analyzed content of hypericins. The lack of correlation between the *HpPR10* gene expression and the presence of dark glands was also confirmed by the similar expression levels of all the *HpPR10* genes in both leaf margin rich with dark glands and leaf interior parts lacking dark glands. Neither in earlier studies the *hyp-1* expression has been found to parallel with the presence of hypericins in the vegetative tissues of *H. perforatum* (Bais et al., 2003; Košuth et al., 2007) or in other species of genus *Hypericum* (Košuth et al., 2011).

Despite of numerous studies of *hyp-1* expression in genus *Hypericum*, the expression has not previously been studied at a cellular level. In the present study, we examined for the first time the expression of *hyp-1* gene in a cellular level by *in situ* RNA localization. The *hyp-1* transcripts in *H. perforatum* stem and root were found to be present in vascular tissues while in leaves the transcripts were also highly associated with mesophyll cells in addition to vascular tissues but not in dark glands. In the vascular tissues, the expression was present in both xylem and phloem cells as well as type A canals. Type A canals have been described earlier for *H. perforatum* by Ciccarelli et al. (2001) but the meaning of the canals for the plant is unknown, although a function in transportation of photosynthates and phloem protectants were suggested. In root and stem, the *hyp-1* expression was highly associated with both xylem and phloem parenchyma cells and companion cells next to sieve elements as well as pericycle cells in root. The parenchyma cells in vascular tissue attend to the lateral transport of compounds, while the pericycle cells are known to be metabolically active and involved in the transport of compounds to and from the vascular bundle that they surround. Our results of the *hyp-1* transcript localization are in agreement with the data obtained by Qian et al. (2012) who studied the cellular location at protein level in *H. perforatum* tissues and found the Hyp-1 protein to be present mainly in vascular tissues of both root and stem as well as in leaf mesophyll with no obvious signal in dark glands.

The expression and location of many PR-10 family proteins of various plant species have been found to be associated with vascular tissues (Breda et al., 1996; Pinto et al., 2005; Kim et al., 2008; Bahramnejad et al., 2010). The biological role of these proteins in the vasculature is not known although defensive role under stress conditions or binding/transportation of hydrophobic ligands have been suggested (Kim et al., 2008; Radauer et al., 2008; Fernandes et al., 2013). The location of the *hyp-1* transcripts in cells of vasculature suggests a similar role. The inconsistent results of *hyp-1* mRNA level with Hyp-1 protein level in *H. perforatum* stem found in the current study can be due to the higher stability of the Hyp-1 protein in stem or indicate the movement of the protein between organs through vasculature. However, the sequences of *H. perforatum* PR-10 proteins, like those of most identified PR-10 proteins, contain no recognizable amino-terminal signal peptide sequence for apoplastic secretion specific to xylem sap proteins (Liu and Ekramoddoullah, 2006; Agarwal and Agarwal, 2014). Since there are suggestions of the role of Hyp-1 in binding and transportation of ligand molecules related to defense and developmental processes (Michalska et al., 2010; Košuth et al., 2013), the possible symplastic mobility of the protein by via plasmodesmata into phloem sap of mature sieve elements needs to be investigated in the future.

Previous studies have evidenced that hypericin biosynthesis is likely to take place in dark glands of *H. perforatum* (Zobayed et al., 2006; Kornfeld et al., 2007; Karppinen et al., 2008; Košuth et al., 2011). However, as discussed above, the *hyp-1* expression does not correlate with hypericin content or presence of dark glands in tissues of *H. perforatum* or other *Hypericum* species. In the present study, equal amounts of the Hyp-1 protein were found in leaf margin and leaf interior parts and, thus, neither our results provide any evidence that the Hyp-1 protein would be specifically associated or accumulating via transportation to the dark glands for its activity in the final stages of hypericin biosynthesis as suggested earlier (Bais et al., 2003). Our results are in agreement with the earlier study of Qian et al. (2012) who reported the presence of the Hyp-1 protein in leaf, stem and root of *H. perforatum* with no association in dark glands in leaves. These findings question the role of the *hyp-1* as a key gene in the hypericin biosynthesis. However, our results cannot exclude the possibility that the Hyp-1 would attend to the biosynthesis/transportation/binding of toxic hypericin (Bais et al., 2003; Michalska et al., 2010) in tissues outside dark glands that also contain minor amounts of hypericins as detected in the current and previous studies (Bais et al., 2002; Gadzovska et al., 2007; Karppinen and Hohtola, 2008; Cui et al., 2010).

Plants are continuously exposed to various stresses in their natural environment. The function of PR-10 proteins is often associated in plant defense because many *PR-10* genes are induced or their expression is up-regulated by different types of biotic and abiotic stresses, such as drought, cold, wounding, and pathogens, as well as stress-related signaling molecules (Pulla et al., 2010; Takeuchi et al., 2011; Agarwal and Agarwal, 2014). Thus, PR-10 family proteins are considered as potentially useful genes for crop improvement. Previously Košuth et al. (2013) described increased expression of *hyp-1* in *H. perforatum* after wounding and treatment with *Agrobacterium* or ABA. ABA-mediated signaling is known to play an important role in plant responses to environmental stresses and plant pathogens (Lee and Luan, 2012). Wounding of plants induces defense responses that resemble those induced by herbivores or pathogen attack. In the current study, we found that all the three newly isolated *PR-10* genes were up-regulated in leaves of *H. perforatum* by wounding as well as by treatment with ABA and SA suggesting a role for the genes in plant defense. The role of SA is established in defense responses against plant pathogens as well as many types of abiotic stresses (Miura and Tada, 2014). In our study, especially the expression of *HpPR10.4* was rapidly and highly induced by SA indicating its special role in SA mediated defense responses. The differential gene expression patterns of the *HpPR10* genes in response to stress-related treatments may imply that they have gene-specific functions under different types of stress conditions. In the present study, *hyp-1* levels were only slightly but not significantly induced by the tested stress treatments which is different to the results of Košuth et al. (2013). The inconsistency in results between the two studies can be due to the differences in the applied treatments (ABA concentration or ABA application method and extent of wounding) or differences in plant material. Depending on the developmental stage of the plant, responses can differ as discussed above.

## Conclusion

We have isolated three previously unidentified *PR-10* family genes from *H. perforatum* and studied their expression along with closely related *hyp-1* in *H. perforatum* tissues and under various stress treatments. Our results show that these genes are constitutively but differently expressed in various *H. perforatum* tissues and their expression is also variably up-regulated by wounding and defense-related signaling molecules. The results suggest a role for these genes in contribution to the defense responses in *H. perforatum* with various functions. Since some *PR-10* genes in other species have been reported to be expressed only in certain specific tissues or under certain stress conditions it cannot be excluded that *H. perforatum* would not have more PR-10 proteins which are to be discovered in the future. Furthermore, the results of the current study do not support the location of the *hyp-1* mRNA or Hyp-1 protein in dark glands or accumulation of the protein via transportation to the dark glands and, thus, question the role of *hyp-1* as a key gene in the hypericin biosynthesis in dark glands of *H. perforatum*.

## Author Contributions

KK and ED performed the analyses. All authors (KK, ED, LJ, and AH) have participated in preparation of the manuscript and have accepted the final version of the manuscript.

## Conflict of Interest Statement

The authors declare that the research was conducted in the absence of any commercial or financial relationships that could be construed as a potential conflict of interest.

## Supplementary material

FIGURE S1 **The contents of hypericins (mg g^-1^ DW) in *H. perforatum* tissues**. Values represent means ± SE of three biological replicates.
