# Supplementary figures and images for: Molecular Cloning and Expression Analysis of hyp-1 Type PR-10 Family Genes in Hypericum perforatum
Source: Front Plant Sci. 2016 Apr 21;7:526. doi: 10.3389/fpls.2016.00526 (PMC4838893; doi:10.3389/fpls.2016.00526)

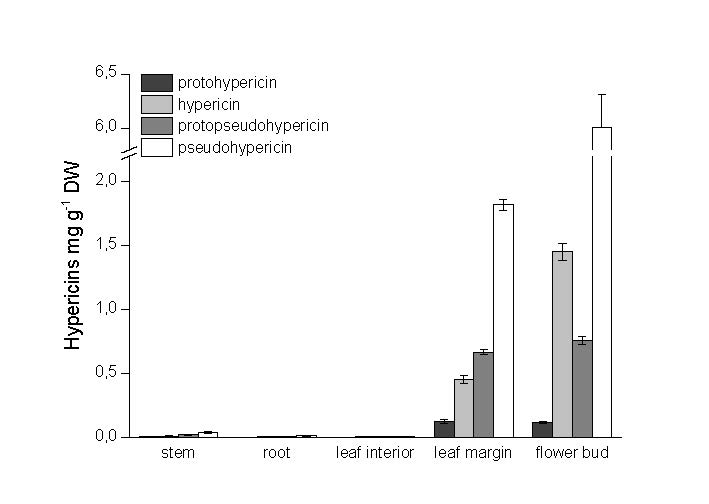

Supplement: FIGURE S1 — The contents of hypericins (mg g-1 DW) in H. perforatum tissues. Values represent means ± SE of three biological replicates. [file Image_1.JPEG]
